# Fatty Acid Methyl Ester (FAME) Succession in Different Substrates as Affected by the Co-Application of Three Pesticides

**DOI:** 10.1371/journal.pone.0145501

**Published:** 2015-12-22

**Authors:** Alessandra Cardinali, Diego Pizzeghello, Giuseppe Zanin

**Affiliations:** Department of Agronomy, Food, Natural resources, Animals and Environment, DAFNAE, University of Padua, Viale dell’Università, 16–35020 Legnaro (PD), Italy; Australian National University, AUSTRALIA

## Abstract

**Introduction:**

In intensive agriculture areas the use of pesticides can alter soil properties and microbial community structure with the risk of reducing soil quality.

**Materials and Methods:**

In this study the fatty acid methyl esters (FAMEs) evolution has been studied in a factorial lab experiment combining five substrates (a soil, two aged composts and their mixtures) treated with a co-application of three pesticides (azoxystrobin, chlorotoluron and epoxiconazole), with two extraction methods, and two incubation times (0 and 58 days). FAMEs extraction followed the microbial identification system (MIDI) and ester-linked method (EL).

**Results and Discussion:**

The pesticides showed high persistence, as revealed by half-life (t_1/2_) values ranging from 168 to 298 days, which confirms their recalcitrance to degradation. However, t_1/2_ values were affected by substrate and compost age down to 8 days for chlorotoluron in S and up to 453 days for epoxiconazole in 12M. Fifty-six FAMEs were detected. Analysis of variance (ANOVA) showed that the EL method detected a higher number of FAMEs and unique FAMEs than the MIDI one, whereas principal component analysis (PCA) highlighted that the monosaturated 18:1ω9*c* and cyclopropane 19:0ω10*c*/19ω6 were the most significant FAMEs grouping by extraction method. The cyclopropyl to monoenoic acids ratio evidenced higher stress conditions when pesticides were applied to compost and compost+soil than solely soil, as well as with final time.

**Conclusion:**

Overall, FAMEs profiles showed the importance of the extraction method for both substrate and incubation time, the t_1/2_ values highlighted the effectiveness of solely soil and the less mature compost in reducing the persistence of pesticides.

## Introduction

Pesticides are widely used in conventional farming systems for plant protection [1,2]. They guarantee high production levels and quality standards, but their toxic effects can extend to non-target soil microorganisms causing changes in microbial community structure and soil quality [3]. The fate of pesticides in soils varies widely, being either destroyed by soil microorganisms over a period of a few days or accumulated steadily from year to year [4]. Soil properties (total organic carbon content, pH, texture, mineralogy and structure), land use and management (crop rotations, pesticide application rate and timing, tillage), and climate play a decisive role in this [5]. Although the soil type and organic carbon content greatly influence the mobility of pesticides through leaching [6], the microbial community has been reported to be usually reduced after the application of pesticides [7,8,9]. A modification in the microbial community structure, towards one more adapted to breaking down complex molecules, has been found to be induced by the addition of three pesticides (i.e., azoxystrobin, chlorotoluron and epoxiconazole) to a compost mixture [10], although compost amendment on its own might induce changes in the soil microbial community [11,12]. Differences in the microbial community structure were also found after the application of azoxystrobin, fludioxonil and penconazole in biobed organic substrates with changes mostly attributed to an inhibition of fungi [13]. More recently, comparing the differences in the metabolism of some pesticides such as terbuthylazine, metribuzin and chlorpyrifos in biobed substrates and soil, it has been reported that the biomixtures mostly stimulated the degradation of terbuthylazine and metribuzin, whereas the soil degraded chlorpyrifos faster [14].

Azoxystrobin (AZO), chlorotoluron (CHL) and epoxiconazole (EPO) are widely used in agriculture [15,16,17]. Azoxystrobin is a broad spectrum fungicide registered for use on over 85 different crops [18]. AZO and its degradation products can be potentially toxic to a wide range of non-target organisms; they can leach through soils for a long period of time following application, thereby posing a potential threat to vulnerable aquatic environments and drinking water resources [19]. CHL is a phenylurea herbicide used in pre and post-emergence of weeds. It is slightly mobile in soil and likely to reach surface waters following field application. CHL is moderately persistent in soil but tends to rapidly disappear in water. EPO is a member of the triazole group of fungicides and acts by inhibiting the biosynthesis of ergosterol, an important component of fungal membranes. It is thus used in the control of diseases caused by Ascomycetes, Basidiomycetes and Deuteromycetes, which mainly affect cereals and oleaginous plants. It may persist in both soil and water systems, causing problems to the soil microbial communities [15].

For a better understanding of the dynamics of microbial community structures after pesticide application it is necessary to elucidate the changes induced in microbial communities that influence the pesticide biodegradation. In a previous study [10], we investigated the effect of three pesticides (azoxystrobin, chlorotoluron and epoxiconazole) on the microbial community structure in a compost biomixture by extracting the fatty acid methyl ester derivatives (FAMEs) profiles with the microbial identification system (MIDI) and ester-linked method (EL). The main results were that FAMEs profile was mostly affected by the extraction method and to a lesser extent by compost age. Thus, the analysis of FAMEs composition might be considered as tool to provide qualitative and quantitative insights into the structure of the microbial community [11,20,21].

In this study, to further our knowledge on the relationship between pesticide persistence and microbial response we worked with the three above-mentioned pesticides with the following objectives: i) to confirm which extracting procedure, MIDI or EL, is more useful to evaluate the effect induced by pesticide application, ii) to investigate whether different substrates (a soil, two different aged composts and their mixture) are reflected in FAMEs profiles, and iii) to look into a relationship between FAMEs and pesticide persistence.

## Materials and Methods

### The trial

This experiment is a lab trial and derive from the co-application of three pesticides in a factorial combination of five types of substrates, hereinafter called soil (S), 3 and 12 aged month compost (3M and 12M) and their mixture with soil (20/80%; v/v) (3M+S and 12M+S), with two extraction methods, and two times of incubation, organized in a randomized block with three replicates.

Our study did not require a statement ethics as not involving human participants and/or tissues, animal research or field trials. This study did not involve endangered or protected species and was conducted entirely by laboratory tests.

### Pesticides

The commercial products Amistar (Syngenta Crop Protection, UK), Alpha Chlortoluron 500 (Makhteshim-Agan, UK) and Opus (BASF, New Zealand) were used, containing 23.1% (wt/wt), 43.9% (wt/wt), and 5.4% (wt/wt) azoxystrobin (AZO) [methyl (*E*)-2-{2-[6-(2-cyanophenoxy)pyrimidin-4-yloxy]phenyl}-3-methoxyacrylate], chlorotoluron (CHL) [3-(3-chloro-4-methylphenyl)-1,1-dimethylurea], and epoxiconazole (EPO) {(2*RS*,3*SR*)-1-[3-(2-chlorophenyl)-2,3-epoxy-2-(4-fluorophenyl)propyl]-1*H*-1,2,4-triazole}, respectively. Pesticide chemical characteristics are reported in S1 Table.

### Compost and soil substrate

The tested substrate was a mixture of urban waste and garden compost from a compost production plant (GESENU) in Pietramelina, Perugia, Italy. Composts aged 3 and 12 months (3M and 12M, respectively) were used. Even if the microbial activity in the early stages of composting is of importance, only 3M and 12M composts were used because this is the age range of industrial composts supplied by production plants and allowed for agricultural use and biobeds. Physical and chemical characteristics of the composts were (*n* = 3, ±standard deviation, SD): pH (H_2_O), 7.8±0.50 and 8.4±0.54; density (g cm^-3^), 0.43±0.09 and 0.57±0.06; OC (%), 30.2±2.20 and 29.0±3.30; N (%), 2.4±0.33 and 3.1±0.70; mineral matter (%), 42±4.20 and 46±3.18 for 3M and 12M, respectively.

The soil, a Calcic Cambisol according to the FAO classification system [22], was sampled from the top 15 cm of an agricultural field of the experimental farm of the University of Padua (Italy). Physical and chemical characteristics of the soil were (*n* = 3, ±SD): textural classification, clay; pH (H_2_O), 7.9±0.25; CaCO_3_ (%), 11.1±1.52; OC (%), 1.2±0.31; CEC (cmol kg^-1^), 25.5±1.80; clay (%), 59.2±3.45; silt (%), 33.5±2.20; sand (%), 7.3±1.12. The two composts and soil were sieved at 2 mm and taken to 60% of their water-holding capacity.

### Degradation study

For the degradation study, one kilogram of soil or compost or soil-compost mixture, in triplicates, was mixed with the three pesticides at the rate of 100 mg of active ingredient in a kg of substrate and incubated in the dark at 20°C. The pesticide rate was selected as being high enough to cause detectable differences in FAME content until the end of the sampling period. Moisture was maintained by addition of the required amount of water at weekly intervals as determined by gravimetric analysis. The treatments were sampled after 0, 1, 3, 7, 14, 21, 32 and 58 days by taking 20 g sub-samples that were each analyzed for pesticides by adding 50 mL of methanol and shaking them for 1 h using a reciprocal shaker. The solutions were separated from the compost by centrifugation (3,490 × *g* for 15 min at 4°C) and analyzed by high-pressure liquid chromatography (HPLC). Percentage recovery rates (means±SD) of the method were 87±6.1 for AZO, 87±8.1 for EPO, and 103±5.8 for CHL. The analysis was performed using an Agilent 1100 series HPLC equipped with a C18, 15 cm × 4.6 mm, 5 μm column. The operating conditions were as follows: solvents, water with 0.1% orthophosphoric acid and acetonitrile (66/34%); flow rate, 1 mL min^-1^; run time, 35 min. Retention times were 7.7, 30.5, and 32.5 min for CHL, AZO, and EPO, respectively. The detection limit was 0.1 μg kg^-1^ for each pesticide. Fatty acid extraction by the EL or MIDI method was done after 0 and 58 days of incubation on 1 g of compost or soil placed in a screw-cap test tube. The procedures used derived from a previous similar study with slight modifications necessary for compost. The EL and MIDI procedures are explained in detail elsewhere [10].

### FAME nomenclature

FAMEs were named in accordance with standard nomenclature and as in reference 10: the total number of carbon atoms, followed by a colon and the number of double bonds. The position of the first double bond is indicated by ω followed by the number of carbon atoms from the aliphatic end. The suffixes *c* and *t* refer to *cis* and *trans* isomers, respectively. Methyl branching at the iso and anteiso positions and that at the 10th carbon atom from the carboxyl end are designated by the prefixes *i*, *a*, and 10*Me*, respectively. The prefix or suffix *cy* denotes cyclopropane fatty acids. When present, the number of hydroxyl substitutions is also given. Selected FAMEs were used as microbial markers according to previous researches [23,24,26] and included gram positive (Gram+) bacteria with iso or anteiso branching, gram negative (Gram‒) bacteria with *cy*17:0, 16:1ω7*c*, 17:1ω7*c*, 18:1ω7*c*, and actinomycetes with 10*Me*16:0, 10*Me*18:0. Fungal markers included saprophytic fungi (18:1ω9*c* and 18:2ω6,9*c*), and arbuscular mycorrhizae (16:1ω5*c*). FAME 20:4ω6,9,12,15*c* was used as a marker for soil microfauna (protozoa and nematodes) and mesofauna [27]. The 16:0 fatty acid is usually considered the most abundant saturated fatty acid in nature and is widely spread among the three microorganism groups. The ratio of the cyclopropyl fatty acids to monoenoic precursors was used as an indicator [25].

### Statistical analysis

Pesticide half-life was calculated according to a first-order kinetic degradation with the module nonlinear estimation of Statistica 7.1 (Statsoft Inc., Tulsa, OK). The dataset is reported in S1 Dataset.

The detection result for each FAME was expressed as a percentage of the total amount of FAMEs. Barlett’s test was used on the data to test the homogeneity of variance. Angular transformation was used when required to normalize the data. FAMEs present in only one replicate of one substrate within the data set were deleted prior to ANOVA; the deleted FAMEs were 10:0 2OH, 10:0 3OH, 12:1, 11:0 2OH, *i*14:1 E, *a*14:0, 13:0 2OH, *i*13:0 3OH, *i*15:1ω9*c*, 16:1ω7*c* alcohol, 16:0 N alcohol, 16:0 2OH, 15:0 2OH, *i*15:0 3OH, *i*17:0ω10*c*, 18:3ω6*c*, *i*17:0 3OH, 19:1ω11*c*/19:1ω9*c*, *i*19:0, *a*19:0, 10*Me*19:0, 18:1 2OH, 18:0 3OH and 20:1ω7*c*. A three-way completely randomized ANOVA was used to compare treatment effects. The factors considered were method, substrate and incubation time. The Student–Newman–Keuls test was applied to compare the differences between group means. To identify the structure of the interdependences between FAMEs, a joint Principal Component Analysis (PCA) was performed on the dataset reported in S2 Dataset. Before PCA, selected FAMEs were obtained by discriminant analysis (data not shown). The standardized variables were submitted to PCA; rotated orthogonal components (varimax rotation method) were extracted and the relative scores were determined. Only Principal Components with eigenvalue >1 were considered for the discussion. ANOVA, discriminant analysis and PCA were performed with SPSS 19 (SPSS, Chicago, IL).

## Results

### Pesticide persistence

The experimental half-life (t_1/2_) values for the three pesticides in the five substrates are reported in Table 1. The degradation process of AZO, CHL and EPO appeared to follow the first-order kinetic equation, C_t_ = C_0_ *e* ^–kt^ where, C_t_ represents the concentration of the pesticide residue at time t; C_0_ represents the initial concentration and k is the rate constant per day. In general, the kinetics showed high coefficients of correlation (*r* ^2^ ranged from 0.83 to 0.97, *p* ≤ 0.001) with t_1/2_ values significantly (*p* ≤ 0.001) affected by pesticide and substrate.

Concerning pesticides, t_1/2_ significantly (*p* ≤ 0.05) decreased on average from EPO (298 days) to AZO (217 days) and CHL (168 days). Regarding substrates, t_1/2_ was significantly (*p* ≤ 0.05) higher in 12M (357 days) than 3M, S and 3M+S (163, 166, 191 days, respectively), while it exhibited an intermediate (*p* ≤ 0.05) value in 12M+S (262 days). The pesticide × substrate interaction was also significant. In particular, t_1/2_ had the highest value in EPO × 12M (453 days) and the lowest in CHL × S (8 days) (*p* ≤ 0.05).

**Table 1:** Half-life (t_1/2_) and *r* ^*2*^ values of the degradation of the three pesticides co-applied in soil (S), compost (M) and their mixture (M+S) (*n* = 3).

	Substrate										
Pesticide	S		3M		12M		3M+S		12M+S		t_1/2_
	t_1/2_	*r* ^*2*^	t_1/2_	*r* ^*2*^	t_1/2_	*r* ^*2*^	t_1/2_	*r* ^*2*^	t_1/2_	*r* ^*2*^	mean
AZO	247*d* *	0.83	114*f*	0.95	317*b*	0.97	150*e*	0.97	258*d*	0.94	217B
CHL	8*g*	0.89	142*e*	0.90	301*b*	0.97	152*e*	0.97	240*d*	0.96	168C
EPO	242*d*	0.83	234*d*	0.95	453*a*	0.86	272*c*	0.96	288*c*	0.93	298A
mean	166c		163c		357a		191c		262b		

AZO, azoxystrobin; CHL, chlorotoluron; EPO, epoxiconazole; 3M, 3 month aged compost; 12M, 12 month aged compost.

*The differences among values were at *p* ≤ 0.05 by Student–Newman–Keuls test.

### FAME analysis

The FAME profiles consisted of 80 fatty acids identified by the two extracting methods, in the five substrates and the two incubation times. Out of these, 56 were consistently present in the samples and used for the data set. These fatty acids ranging in carbon chain length from C9 to C20 consisted of saturated, mono- and polyunsaturated, branched, hydroxy, methylated, cyclopropane and mixed fatty acids. The percentage of FAMEs was variable across detections and ranged from 0.03 to 100% whereas the variability between replicates was very low.

Changes in the community structure determined by ANOVA in response to extraction method, substrate and incubation time were all significant (*p* ≤ 0.001).

The EL method gave a higher number of detections than MIDI (1,301 and 405). There were 54 and 28 FAMEs for EL and MIDI methods, respectively. Twenty-six were detected by both methods, while 28 and 2 were found only in EL and MIDI, the so-called unique FAMEs. Within the common fatty acids (Table 2), monounsaturated 18:1ω9*c*, 18:1ω7*c*/18:1ω6*c*, 18:1ω5*c* and 16:1ω5*c*, saturated 18:0, polyunsaturated *a*18:0/18:2ω6,9*c* and branched *i*14:0, *i*15:0, *a*15:0 and *i*16:0 were high (*p* ≤ 0.05) in EL, whereas cyclopropane *cy*19:0ω10*c*/19ω6, monounsaturated 16:1ω7*c*/16:1ω6*c*, saturated 12:0, hydroxy 18:0 2OH and 12:0 3OH and branched *a*13:0 and *i*20:0 were high (*p* ≤ 0.05) in MIDI. Among the unique FAMEs (S2 Table), EL showed a slight presence of saturated 20:0, 17:0 and 10:0, branched *a*17:0 and *i*17:0, cyclopropane *cy*19:0 ω8*c*, whereas MIDI had noticeable amounts of monounsaturated 15:1ω8*c* and 17:1ω7*c*. Methylated FAMEs were present only in EL method.

**Table 2:** Common FAMEs grouped by extraction method: microbial identification system (MIDI) and ester-linked procedure (EL) expressed as a percentage (*n* = 30).

FAMEs		METHOD	
Type	Name	MIDI	EL
Saturated	12:0	5.73a	1.46b
	18:0	0.66b	5.28a
Monounsaturated	16:1ω5*c*	0.41b	1.24a
	16:1ω7*c*/16:1ω6*c*	8.29a	3.39b
	18:1ω7*c*/18:1ω6*c*	0.46b	4.69a
	18:1ω9*c*	5.21b	17.79a
	18:1ω5*c*	1.55b	4.28a
Polyunsaturated	*a*18:0/18:2ω6,9*c*	1.21b	4.92a
Branched	*a*13:0	2.17a	0.20b
	*i*14:0	0.15b	1.18a
	*i*15:0	1.35b	3.42a
	*a*15:0	1.77b	4.49a
	*i*16:0	0.14b	3.54a
	*i*20:0	0.74a	0.12b
Hydroxy	12:0 3OH	0.57a	0.03b
	18:0 2OH	2.31a	0.26b
Cyclopropane	*cy*19:0ω10*c*/19ω6	30.95a	0.47b
Mixed	*a*15:1 A	0.13b	0.87a
	*a*17:1 B/*i*17:1 I	1.05a	0.78b

Different letters in the same row indicate differences at *p* ≤ 0.05 by the Student-Newman-Keuls test.

Within the substrates, 12M+S and S gave the highest (398) and lowest (204) number of detections (*p* ≤ 0.05), whereas 3M, 12M, 3M+S and 12M+S had more FAMEs (*ca* 55) than S (32) (*p* ≤ 0.05). S profile was dominated by the saturated 16:0 (Table 3), whereas 3M, 12M, 3M+S and 12M+S profiles were dominated by the cyclopropane *cy*19:0ω10*c*/19ω6 (S3 Table) and monounsaturated 18:1ω9*c* (Table 3). Among other common FAMEs (Table 3), the monounsaturated 18:1ω7*c*/18:1ω6*c* and 16:1ω5*c*, polyunsaturated *a*18:0/18:2ω6,9*c*, mixed *i*12:0 3OH, and *i*11:0 3OH, branched *i*15:0, *a*13:0 and *i*14:0, saturated 13:0, hydroxyl 11:0 3OH and methylated 10*Me*16:0 were significantly (*p* ≤ 0.05) higher in S than in 3M, 12M, 3M+S and 12M+S (Table 3).

**Table 3:** Common FAMEs grouped by substrate: soil (S), 3 and 12 month aged compost (3M and 12M) and their mixture (3M+S and 12M+S) expressed as a percentage (*n* = 12).

FAME		SUBSTRATE				
Type	Name	S	3M	12M	3M+S	12M+S
Saturated	12:0	2.69b	3.77a	3.79a	4.12a	3.60a
	13:0	2.28a	0.51c	0.51c	1.16b	0.75c
	14:0	2.91a	2.08ab	1.49b	1.59b	2.15ab
	16:0	35.38a	12.20b	11.23b	12.12b	11.11b
	18:0	1.79d	3.49a	3.18b	2.73c	3.65a
	20:0	0.91a	0.76b	0.52c	0.71b	0.51c
Monounsaturated	16:1ω5*c*	2.22a	0.60c	0.25d	0.74b	0.34d
	16:1ω7*c*/16:1ω6*c*	4.79b	6.09a	6.55a	5.84a	5.93a
	17:1ω8*c*	0.82a	0.23b	0.23b	0.26b	0.25b
	18:1ω7*c*/18:1ω6*c*	4.70a	2.28b	1.89c	2.08bc	1.92c
	18:1ω9*c*	6.77d	12.27bc	13.88a	11.41c	13.14ab
	20:1ω9*c*	0.67b	0.45c	1.12a	0.40c	1.16a
Polyunsaturated	*a*18:0/18:2ω6,9*c*	3.61a	3.21b	2.43c	3.00b	3.07b
Branched	*a*13:0	2.22a	0.41d	1.12b	1.37b	0.80c
	*i*14:0	1.14a	0.38c	0.46c	0.46c	0.88b
	*i*15:0	3.59a	1.61d	2.27c	1.74d	2.70b
	*a*15:0	3.05b	2.84b	3.71a	2.43c	3.63a
	*i*16:0	1.66c	2.03a	1.75c	1.87b	1.90b
	*i*17:0	0.76a	0.74a	0.58b	0.76a	0.60b
	*a*17:0	1.06c	1.32b	1.40a	1.32b	1.38ab
Hydroxy	11:0 3OH	2.25a	0.12d	0.62c	0.96b	0.64c
Methylated	10*Me*16:0	2.09a	0.74c	0.82bc	0.87b	0.88b
	11*Me*18:1ω7*c*	0.42a	0.17b	0.08c	0.15b	0.10c
	10*Me*18:0, TBSA	0.35c	0.75b	1.34a	0.66b	1.35a
Cyclopropane	*cy*17:0	0.43ab	0.46ab	0.41b	0.47ab	0.48a
	*cy*19:0ω8*c*	1.33a	1.25a	0.94bc	1.06b	0.81c
Mixed	*i*11:0 3OH	1.15a	0.07d	0.05d	0.67b	0.36c
	*i*12:0 3OH	3.62a	0.47c	0.57c	1.22b	0.88bc
	*a*17:1 B/*i*17:1 I	1.47a	0.52b	0.79b	0.51b	1.31a

Different letters in the same row indicate differences at *p* ≤ 0.05 by the Student-Newman-Keuls test.

On the contrary, the monounsaturated 16:1ω7*c*/16:1ω6*c* and saturated 12:0 were significantly (*p* ≤ 0.05) higher in 3M, 12M, 3M+S and 12M+S than in S. Along with the unique FAMEs (S3 Table), the monounsaturated 18:1ω5*c*, 17:1ω7*c* and 15:1ω8*c* were present in 3M, 12M, 3M+S, 12M+S whereas they were absent in S. The saturated 10:0 and 17:0; monounsaturated 16:1ω9*c*; polyunsaturated 20:4ω6,9,12,15*c*; branched *i*18:0; hydroxy 12:0 3OH and 17:0 3OH; mixed *i*15:1 G, *a*15:1 A, *i*16:1 G, *a*17:1 A and *i*19:1 I were found in 3M, 12M, 3M+S and 12M+S.

Concerning the incubation time, the initial (day 0) and final time (58 days) differed (*p* ≤ 0.05) in the number of detections (915 versus 791, respectively). Out of the 54 common FAMEs, 14 differed in amount. In particular (Table 4 and S4 Table), the saturated 12:0 and 13:0, the branched: *a*13:0, *i*14:0 and *i*20:0, the hydroxy 11:0 3OH and the mixed *i*12:0 3OH were significantly (*p* ≤ 0.05) higher at day 0 than 58 days, whereas the cyclopropane *cy*19:0ω8*c*, the methylated 10*Me*17:0 and 11*Me*18:1ω7*c*, prevailed (*p* ≤ 0.05) at 58 days.

**Table 4:** Common FAMEs grouped by incubation time (0 and 58 days) expressed as a percentage (*n* = 30).

FAMEs		IT	
Type	Name	0	58
Saturated	10:0	0.17a	0.05b
	12:0	4.54a	2.65b
	13:0	2.05a	0.03b
Branched	*i*11:0	0.04a	0.01b
	*a*13:0	2.22a	0.15b
	*i*14:0	0.88a	0.44b
	*i*20:0	0.78a	0.08b
Hydroxy	11:0 3OH	1.79a	0.05b
Methylated	10*Me*17:0	0.16b	0.31a
	11*Me*18:1ω7*c*	0.04b	0.33a
Cyclopropane	*cy*19:0ω8*c*	0.71b	1.45a
Mixed	*i*12:0 3OH	2.69a	0.02b
	*a*15:1 A	0.70a	0.30b
	*i*19:1 I	0.51a	0.07b

Different letters in the same row indicate differences at *p* ≤ 0.05 by the Student-Newman-Keuls test.

### PCA analysis

**Fig 1:**
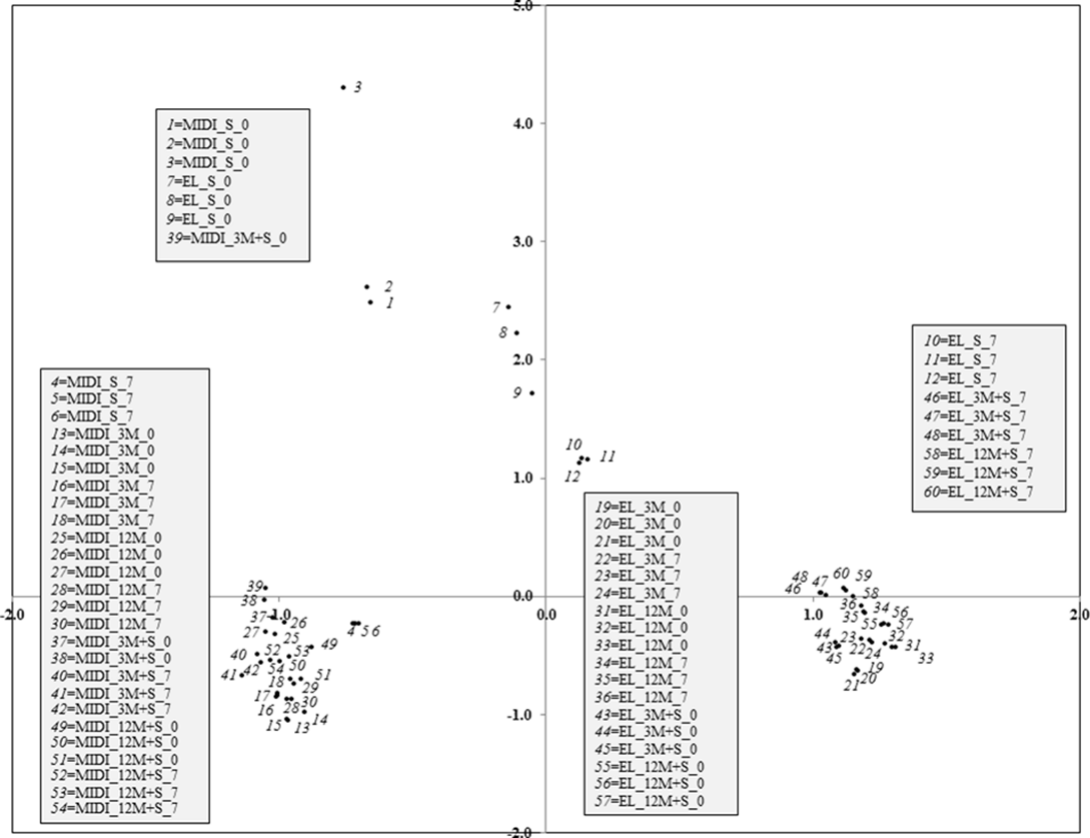
Position of the samples from PCA analysis. Position of the samples by method of extraction (microbial identification system, MIDI; ester-linked, EL), substrate (soil, S; compost aged 3 months, 3M; compost aged 12 months, 12M; compost aged 3 months mixed with soil, 3M+S; compost aged 12 months mixed with soil, 12M+S) and incubation time (0 and 58 days) in the reduced space of the first two principal components analysis (58.75% and 16.05% of the variance, respectively) (*n* = 3).

PCA identified the fatty acids that were important in explaining the variability in the FAME profiles. PCA extracted three factors accounting for 83.91% of the total variance. Factor 1 (*eigenvalue* = 23.5) explained 58.75% of the variance and was very highly (> 0.9) correlated (S5 Table) with 10:0, 14:0, 15:1ω6*c*, *i*16:1 G, 16:1ω5*c*, 16:0, *a*17:1 B/*i*17:1 I, *a*17:1 A, *i*17:0, *a*17:0, 17:1ω7*c*, *cy*17:0, 10*Me*17:0, *a*18:0/18:2ω6,9*c*, 18:0, 11*Me*18:1ω7*c*, *i*19:1 I, 17:0 3OH, *cy*19:0ω10*c*/19ω6, *cy*19:0ω8*c*, 19:0, 18:0 2OH. Factor 2 (*eigenvalue* = 6.42) accounted 16.05% and highly (> 0.8) correlated (S5 Table) with 13:0 and *i*11:0 3OH, and (approx. 0.7) with 12:0, *i*14:0, *a*13:0 and *i*13:0. Factor 3 (*eigenvalue* = 3.64) explained the remaining 9.11% and correlated (S4 Table) with 16:1ω7*c*/16:1ω6*c*, 10*Me*18:0 TBSA and *i*20:0. Plotting data according to PC1 and PC2 (Fig 1) identified two main clusters with narrow points mainly distributed along axis 1, while few points were distributed along axis 2. In general, axis 1 high values corresponded to the EL method, whereas axis 1 low values characterized MIDI. Fig 1 also shows that S was more widely distributed along axis 2 while 3M, 12M, 3M+S and 12M+S resulted in two narrow clusters. Concerning incubation time, both day 0 and 58 days scattered in all three clusters. Considering all three factors together it is interesting to note that MIDI × 58 days has lower values in both axis 1 and 2 (sector III) than MIDI × day 0 (sector III and IV). Moreover, S × MIDI × day 0 and S × EL × day 0 are in the same sector IV while S × EL × 58 days and S × MIDI × 58 days have opposite values (sector I and III, respectively).

## Discussion

The three studied pesticides showed high persistence (t_1/2_ values from 168 to 298 days) which is consistent with the literature and confirms their recalcitrance to degradation [4,28]. t_1/2_ values resulted as being strongly affected by substrate type. In fact, the pesticide half-life reduces 2.1 fold when comparing 12M with S. Pesticide degradation also depended on the compost age. Indeed, persistence decreased with the reduction in compost age, as resulted from comparing 12M with 3M, in accordance with our previous work [10]. However, if less mature compost is better able than mature compost to degrade pesticides, probably due to the greater carbon sources for microorganisms [29], the abundance of functional groups in a mature compost might increase the number of interactions with a pesticide and thus its persistence, as shown between a humic acid and a herbicide [30]. From these considerations, it is likely that 12M+S substrate showed an intermediate t_1/2_ value between 12M and S. Note that the half-life varied to a greater extent when the interaction between pesticide and substrate was considered. In fact, the t_1/2_ value lowered to 8 days for CHL in S, whereas it increased up to 453 days for EPO in 12M. These results strongly highlight the importance of substrate and compost age as factors in determining pesticide persistence.

Qualitative differences in FAMEs extracted by the two methods should be considered when determining which method to use. We found that the abundances of several important marker FAMEs were dependent on the extraction method (Fig 1 and S1 Fig). For example, 18:1ω9*c* and 16:1ω5*c* may be marker for saprophytic and arbuscular mycorrhizal fungi [23] and in agricultural soils their relative amount might be doubled when the EL method was used. Conversely, the actinomycetes marker 10*Me*16:0 and 10*Me*18:0 [23,24] was more abundant when soils were extracted with the EL method. Also, abundances of several markers for Gram-negative bacteria differed between the two extraction methods. Relative amounts of hydroxylated and cyclopropane FAMEs were greater in MIDI extracts, whereas EL extracts contained relatively greater amounts of iso and anteiso branching FAMEs. This is consistent with the results found previously in compost [10,31,32]. Overall, our results confirmed that EL had a higher number of distinguishing FAMEs than MIDI. This is probably due to the fact that MIDI is not specifically designed for the maximum number of FAME but is adjusted more to groundwater microbial communities [33]. MIDI also considers free fatty acids in contrast to the EL method which extracts ester-linked fatty acids. Differences between the two methods might also be due to FAME losses during saponification and the following incubation that is done with the MIDI method [31,34,35]. Moreover, both methods are non-specific analyses and fatty acids from phospholipids, glycolipids and neutral fats are extracted from intact microbial cells as well as from dead organic material. Nevertheless, the high number of FAMEs in the EL method is a response to the greater sensitivity than the MIDI one and seems to ensure a good description of the microbial community structure in both compost and soil.

EL and MIDI methods also showed a ratio of cyclopropyl to monoenoic acids from very low (0.11) to high (1.28). High values of this ratio are generally indicative of stress conditions, but in this context seem to confirm that the two methods extract a different microbial population [10,31]. In line with this hypothesis, the high presence in EL extracts of iso and anteiso branched FAMEs (i.e., *i*16:0, *a*16:0, *i*17:0, *a*17:0), biomarkers for Gram-positive bacteria, methylated, biomarkers for actinomycetes (i.e., 10*Me*16:0, 10*Me*17:0, 11*Me*18:1ω7*c*), and *a*18:0/18:2ω6,9*c*, biomarker for fungi, highlight high biological activity and microbial growth.

Concerning substrates, the FAMEs well distinguished S from all the others. Variation of S from the other substrates was along axis 2 of the PCA (Fig 1), which correspond to the importance of the saturated 13:0 and the branched *i*14:0 and *a*13:0 (S1 Fig). On the contrary, variations versus 3M, 12M, 3M+S and 12M+S were mainly along axis 1, with differences in the cyclopropane *cy*19:0ω10*c*/19ω6 and monounsaturated 18:1ω9*c* (Fig 1 and S1 Fig). Differently from the PCA, ANOVA also showed the higher presence of the saturated 16:0 in S than in the other substrates. Dominance of saturated and monounsaturated fatty acids was previously found in other treated soils and compost [10,36]. These results highlight that organic matter is an important factor governing the composition of microbial communities [25,37]. From the cyclopropyl to monoenoic acids ratio, S exhibited a lower value (0.09) than all the other substrates (approx. 0.67). This demonstrates higher stress conditions when pesticides were applied to compost and compost+soil than when applied to solely soil. Indeed, monoenoic acids are considered to be associated with high carbon availability, whereas cyclopropane is produced under limited carbon source [38,39].

Slight but significant differences were also found in FAMEs percentage between compost and compost+soil. In fact, the monounsaturated 18:1ω9*c* and branched *i*14:0, *i*15:0, *a*15:0, *a*17:0 showed increases from substrate 3M to 12M and from 3M+S to 12M+S. This is in agreement with the increases of Gram-negative bacteria and fungi reported over time during composting [10,11,36,40].

FAMEs were also affected by incubation time. In our previous data [10] the content of mixed FAMEs decreased regularly as time increased, while those of saturated, branched and methylated FAMEs fluctuated, when time was intermediate. In the actual study, initial time resulted more sensitive than final time. In particular saturated, branched, hydroxy FAMEs decreased over time, whilst methylated and cyclopropane FAMEs increased. These differences might confirm the relationship between substrate and microbial succession which determine a different pattern in the fatty acids over time [32]. The ratio cyclopropyl to monoenoic acids revealed somewhat differences between the two incubation time with a decreasing value from day 58 to day 0 (0.65 and 0.53). The importance of fatty acids 10*Me*17:0 and 10*Me*18:1ω7*c* at day 58 may be partially explained by the increased growth of actinomycetes, since 10*Me*17:0 and 10*Me*18:1ω7*c* are signature for this bacterial group. On the other hand, the conversion of monounsaturated like 16:1ω5*c* and 18:1ω9*c* to cyclopropyl 19:0ω10*c*/19ω6 is an indication of a move from the logarithmic growth phase, in Gram-negative bacteria [32]. Thus, the incubation is likely traduced with an increasing of stress condition [41].

In conclusion, from these results it is likely that pesticides degradation might be strongly affected by the type of substrate in which they are applied. Indeed, the soil and less mature compost seem to be the most active in reducing the degradation time of the three pesticides. Concerning the microbial community, the FAMEs, resulting from the pesticides addition in the five substrates, were strongly influenced by the extracting methods. High sensitivity of EL confirms the use of this method for a wider description of the microbial community, which in turn may give more information on the pesticides degradation than MIDI. Substrates and incubation time also altered the FAMEs profile. In particular, the monounsaturated and the cyclopropyl fatty acids resulted as being important in distinguishing among methods, substrates and incubation time. Moreover the lowest stress conditions of the microbial community found in soil and at the initial incubation time degraded the pesticides best.

## Supporting Information

S1 Dataset Dataset for pollution degradation. (XLSX)

S2 Dataset Dataset for PCA. (XLSX)

S1 Fig Variables from PCA result. Variables projected in the plane determined by the first two principal axes (58.75% and 16.05% of the variance, respectively). In the boxes correspondence between label and name of fatty acid follows the y ordinate.
(DOCX)

S1 Table Selected chemical properties and environmental parameters of the three pesticides (from PPDB, 2015). (DOCX)

S2 Table Unique FAMEs and non-significant FAMEs grouped by extraction method: microbial identification system (MIDI) and ester-linked procedure (EL) expressed as a percentage (*n* = 30). (DOCX)

S3 Table Uncommon FAMEs grouped by substrate: soil (S), 3 and 12 month aged compost (3M and 12M) and their mixture (3M+S and 12M+S) expressed as a percentage (*n* = 12). (DOCX)

S4 Table Non-significant FAMEs grouped by incubation time (0 and 58 days) expressed as a percentage (*n* = 30). (DOCX)

S5 Table Loadings values of the selected FAMEs on the axes identified by principal components analysis for five substrates treated with three pesticides and followed degradation time. (DOCX)

## Abbreviations

AZO: Azoxystrobin
CHL: Chlorotoluron
EPO: Epoxiconazole
EL: Ester-linked method
FAMEs: Fatty acid methyl esters
t_1/2_: Half-life
MIDI: Microbial identification system
