# Supplementary material for: Fatty Acid Methyl Ester (FAME) Succession in Different Substrates as Affected by the Co-Application of Three Pesticides
Source: PLoS One. 2015 Dec 22;10(12):e0145501. doi: 10.1371/journal.pone.0145501 (PMC4687828; doi:10.1371/journal.pone.0145501)
Supplement: S1 Fig — Variables projected in the plane determined by the first two principal axes (58.75% and 16.05% of the variance, respectively). In the boxes correspondence between label and name of fatty acid follows the y ordinate. (DOCX) [file pone.0145501.s003.docx]

**S1 Figure**

Variables from PCA result. Variables projected in the plane determined by the first two principal axes (58.75% and 16.05% of the variance, respectively). In the boxes correspondence between label and name of fatty acid follows the y ordinate.
