# Supplementary material for: Fatty Acid Methyl Ester (FAME) Succession in Different Substrates as Affected by the Co-Application of Three Pesticides
Source: PLoS One. 2015 Dec 22;10(12):e0145501. doi: 10.1371/journal.pone.0145501 (PMC4687828; doi:10.1371/journal.pone.0145501)
Supplement: S1 Table — (DOCX) [file pone.0145501.s004.docx]

**S1 Table**

Selected chemical properties and environmental parameters of the three pesticides (from PPDB, 2015)

| Properties | Unit | Azoxystrobin  (AZO) | Chlorotoluron  (CHL) | Epoxiconazole  (EPO) |  |
| --- | --- | --- | --- | --- | --- |
| Molecular weight |  | 403.4 | 212.7 | 329.8 |  |
| Water solubility at 20 °C | mg L^-1^ | 6.7 | 74 | 7.1 | |
| Melting point | °C | 116 | 148.1 | 136.7 |  |
| Freundlich constant (*Kf*) |  | 7.4 | 5.2 | 4.8-21.8 |  |
| K_oc_ | mL g^-1^ | 304-739 | 108-384 | 957-2647* | |
| t_1/2_ (lab at 20 °C) | days | 35-248 | 52-66 | 98-649 |  |

*MacBean C, The Pesticide Manual, 16th edition. British Crop Production Council, Alton, Hampshire, UK (2012).

Pesticide Properties Database (PPDB), 2015. University of Hertfordshire as part of the EU-funded FOOTPRINT project (FP6-SSP-022704). Available at: http://www.eu-footprint.org/ppdb.html). Accessed 15 January 2015.
