# Supplementary material for: Fatty Acid Methyl Ester (FAME) Succession in Different Substrates as Affected by the Co-Application of Three Pesticides
Source: PLoS One. 2015 Dec 22;10(12):e0145501. doi: 10.1371/journal.pone.0145501 (PMC4687828; doi:10.1371/journal.pone.0145501)
Supplement: S2 Table — (DOCX) [file pone.0145501.s005.docx]

**S2 Table**

Unique FAMEs and non-significant FAMEs grouped by extraction method: microbial identification system (MIDI) and ester-linked procedure (EL) expressed as a percentage (*n* = 30)

| FAMEs |  |  | METHOD | |
| --- | --- | --- | --- | --- |
| Type | Name |  | MIDI | EL |
| Saturated | 9:0 |  | n.d. | 0.03 |
|  | 10:0 |  | n.d. | 0.21 |
|  | 11:0 |  | n.d. | 0.06 |
|  | 13:0 |  | 1.40 | 0.68 |
|  | 14:0 |  | 2.03 | 2.06 |
|  | 16:0 |  | 16.54 | 16.27 |
|  | 17:0 |  | n.d. | 0.49 |
|  | 19:0 |  | n.d. | 0.14 |
|  | 20:0 |  | n.d. | 1.36 |
| Monounsaturated | 15:1ω6*c* |  | n.d. | 0.11 |
|  | 15:1ω8*c* |  | 4.27 | n.d. |
|  | 16:1ω9*c* |  | n.d. | 0.30 |
|  | 17:1ω7*c* |  | 4.06 | n.d. |
|  | 17:1ω8*c* |  | n.d. | 0.72 |
|  | 20:1ω9*c* |  | n.d. | 1.52 |
| Polyunsaturated | 20:4ω6,9,12,15*c* |  | n.d. | 0.18 |
| Branched | *i*11:0 |  | n.d. | 0.05 |
|  | *i*13:0 |  | n.d. | 0.07 |
|  | *a*16:0 |  | n.d. | 0.08 |
|  | *i*17:0 |  | n.d. | 1.38 |
|  | *a*17:0 |  | n.d. | 2.60 |
|  | *i*18:0 |  | n.d. | 0.39 |
| Hydroxy | 11:0 3OH |  | 1.20 | 0.63 |
|  | 17:0 3OH |  | n.d. | 0.15 |
| Methylated | 10*Me*16:0 |  | n.d. | 2.16 |
|  | 10*Me*17:0 |  | n.d. | 0.47 |
|  | 11*Me*18:1ω7*c* |  | n.d. | 0.37 |
|  | 10*Me*18:0, TBSA |  | n.d. | 1.78 |
| Cyclopropane | *cy*17:0 |  | n.d. | 0.90 |
|  | *cy*19:0ω8*c* |  | n.d. | 2.16 |
| Mixed | *i*11:0 3OH |  | 0.59 | 0.33 |
|  | *i*12:0 3OH |  | 1.79 | 0.91 |
|  | *i*15:1 G |  | n.d. | 0.32 |
|  | *i*16:1 G |  | n.d. | 0.35 |
|  | *a*17:1 A |  | n.d. | 0.30 |
|  | 16:1 2OH |  | n.d. | 0.61 |
|  | *i*19:1 I |  | 0.23 | 0.34 |

n.d. = not detected.
