# Supplementary material for: Fatty Acid Methyl Ester (FAME) Succession in Different Substrates as Affected by the Co-Application of Three Pesticides
Source: PLoS One. 2015 Dec 22;10(12):e0145501. doi: 10.1371/journal.pone.0145501 (PMC4687828; doi:10.1371/journal.pone.0145501)
Supplement: S3 Table — (DOCX) [file pone.0145501.s006.docx]

**S3 Table**

Uncommon FAMEs grouped by substrate: soil (S), 3 and 12 month aged compost (3M and 12M) and their mixture (3M+S and 12M+S) expressed as a percentage (*n* = 12)

| FAME |  |  | SUBSTRATE | | | | |
| --- | --- | --- | --- | --- | --- | --- | --- |
| Type | Name |  | S | 3M | 12M | 3M+S | 12M+S |
| Saturated | 9:0 |  | n.d. | 0.03a | 0.01b | 0.02b | 0.03a |
|  | 10:0 |  | n.d. | 0.12b | 0.10b | 0.18a | 0.14b |
|  | 11:0 |  | n.d. | 0.03b | 0.02b | 0.07a | 0.02b |
|  | 17:0 |  | n.d. | 0.34a | 0.27b | 0.34a | 0.28b |
|  | 19:0 |  | n.d. | 0.08a | 0.09a | 0.07a | 0.10a |
| Monounsaturated | 15:1ω6*c* |  | n.d. | 0.08a | 0.05a | 0.08a | 0.06a |
|  | 15:1ω8*c* |  | n.d. | 3.28a | 1.63b | 2.84a | 2.91a |
|  | 16:1ω9*c* |  | n.d. | 0.21b | 0.14d | 0.24a | 0.17c |
|  | 17:1ω7*c* |  | n.d. | 2.57a | 2.66a | 2.51a | 2.40a |
|  | 18:1ω5*c* |  | n.d. | 3.27c | 4.41b | 5.32a | 1.58d |
| Polyunsaturated | 20:4ω6,9,12,15*c* |  | n.d. | 0.12b | 0.04c | 0.21a | 0.09b |
| Branched | *i*11:0 |  | n.d. | 0.03a | 0.03a | 0.03a | 0.03a |
|  | *i*13:0 |  | n.d. | 0.05b | 0.04b | 0.03c | 0.05a |
|  | *a*16:0 |  | n.d. | 0.06a | 0.05a | 0.03b | 0.05a |
|  | *i*18:0 |  | n.d. | 0.32a | 0.18b | 0.30a | 0.16b |
|  | *i*20:0 |  | n.d. | 1.14a | 0.10c | 0.89b | n.d. |
| Hydroxy | 12:0 3OH |  | n.d. | 0.03d | 0.68a | 0.28c | 0.50b |
|  | 17:0 3OH |  | n.d. | 0.12a | 0.12a | 0.05bc | 0.07ab |
|  | 18:0 2OH |  | 0.49c | n.d. | n.d. | 2.47b | 3.46a |
| Methylated | 10*Me*17:0 |  | 0.26 | 0.28 | 0.21 | 0.22 | 0.20 |
| Cyclopropane | *cy*19:0ω10*c*/19ω6 |  | n.d. | 19.94ab | 20.91a | 18.57b | 19.13ab |
| Mixed | *i*15:1 G |  | n.d. | 0.13c | 0.26b | 0.12c | 0.30a |
|  | *a*15:1 A |  | n.d. | 0.33c | 0.74b | 0.33c | 1.08a |
|  | *i*16:1 G |  | n.d. | 0.21a | 0.21a | 0.23a | 0.23a |
|  | *a*17:1 A |  | n.d. | 0.13c | 0.23a | 0.16b | 0.24a |
|  | 16:1 2OH |  | 1.42a | n.d. | 0.03b | 0.04b | 0.03b |
|  | *i*19:1 I |  | n.d. | 0.94a | 0.11c | 0.33b | 0.06c |

Different letters in the same row indicate differences at P≤0.05 by the Student-Newman-Keuls test. n.d. = not detected.
