# Supplementary material for: Fatty Acid Methyl Ester (FAME) Succession in Different Substrates as Affected by the Co-Application of Three Pesticides
Source: PLoS One. 2015 Dec 22;10(12):e0145501. doi: 10.1371/journal.pone.0145501 (PMC4687828; doi:10.1371/journal.pone.0145501)
Supplement: S4 Table — (DOCX) [file pone.0145501.s007.docx]

**S4 Table**

Non-significant FAMEs grouped by incubation time (0 and 58 days) expressed as a percentage (*n* = 30)

| FAMEs |  |  | IT^†^ | |  |
| --- | --- | --- | --- | --- | --- |
| Type | Name |  | 0 | | 58 |
| Saturated | 9:0 |  | | 0.02 | 0.01 |
|  | 11:0 |  | 0.06 | | n.d. |
|  | 14:0 |  | 2.16 | | 1.93 |
|  | 16:0 |  | 11.79 | | 21.02 |
|  | 17:0 |  | 0.22 | | 0.27 |
|  | 18:0 |  | 2.86 | | 3.08 |
|  | 19:0 |  | 0.09 | | 0.04 |
|  | 20:0 |  | 0.60 | | 0.76 |
| Monounsaturated | 15:1ω6*c* |  | 0.06 | | 0.06 |
|  | 15:1ω8*c* |  | 1.72 | | 2.54 |
|  | 16:1ω9*c* |  | 0.17 | | 0.13 |
|  | 16:1ω5*c* |  | 0.78 | | 0.87 |
|  | 16:1ω7*c*/16:1ω6*c* |  | 6.16 | | 5.52 |
|  | 17:1ω7*c* |  | 1.91 | | 2.15 |
|  | 17:1ω8*c* |  | 0.35 | | 0.36 |
|  | 18:1ω7*c*/18:1ω6*c* |  | 2.41 | | 2.75 |
|  | 18:1ω9*c* |  | 12.06 | | 10.93 |
|  | 18:1ω5*c* |  | 2.64 | | 3.19 |
|  | 20:1ω9*c* |  | 0.59 | | 0.93 |
| Polyunsaturated | *a*18:0/18:2ω6,9*c* |  | 3.62 | | 2.51 |
|  | 20:4ω6,9,12,15*c* |  | 0.05 | | 0.13 |
| Branched | *i*13:0 |  | 0.03 | | 0.04 |
|  | *i*15:0 |  | 2.31 | | 2.45 |
|  | *a*15:0 |  | 3.26 | | 3.00 |
|  | *i*16:0 |  | 1.98 | | 1.70 |
|  | *a*16:0 |  | 0.05 | | 0.03 |
|  | *i*17:0 |  | 0.64 | | 0.74 |
|  | *a*17:0 |  | 1.34 | | 1.25 |
|  | *i*18:0 |  | 0.20 | | 0.19 |
| Hydroxy | 12:0 3OH |  | 0.47 | | 0.13 |
|  | 17:0 3OH |  | 0.10 | | 0.05 |
|  | 18:0 2OH |  | 0.74 | | 1.83 |
| Methylated | 10*Me*16:0 |  | 0.86 | | 1.30 |
|  | 10*Me*18:0, TBSA |  | 1.13 | | 0.64 |
| Cyclopropane | *cy*17:0 |  | 0.44 | | 0.46 |
|  | *cy*19:0ω10*c*/19ω6 |  | 14.21 | | 17.21 |
| Mixed | *i*11:0 3OH |  | 0.92 | | n.d. |
|  | *i*15:1 G |  | 0.18 | | 0.15 |
|  | *i*16:1 G |  | 0.20 | | 0.15 |
|  | *a*17:1 B/*i*17:1 I |  | 0.66 | | 1.18 |
|  | *a*17:1 A |  | 0.18 | | 0.13 |
|  | 16:1 2OH |  | 0.27 | | 0.34 |

n.d. = not detected.
