# Supplementary material for: Fatty Acid Methyl Ester (FAME) Succession in Different Substrates as Affected by the Co-Application of Three Pesticides
Source: PLoS One. 2015 Dec 22;10(12):e0145501. doi: 10.1371/journal.pone.0145501 (PMC4687828; doi:10.1371/journal.pone.0145501)
Supplement: S5 Table — (DOCX) [file pone.0145501.s008.docx]

**S5 Table**

Loadings values of the selected FAMEs on the axes identified by principal components analysis for five substrates treated with three pesticides and followed degradation time

|  | PCs | |  | |
| --- | --- | --- | --- | --- |
| FAMEs | 1 | 2 | | 3 |
| 9:0 | 0.779 | -0.190 | | -0.227 |
| 10:0 | 0.941 | -0.197 | | -0.204 |
| *i*11:0 | 0.783 | -0.206 | | -0.284 |
| 11:0 | -0.855 | 0.002 | | -0.221 |
| 12:0 | -0.250 | 0.773 | | -0.457 |
| *i*11:0 3OH | -0.195 | 0.840 | | -0.457 |
| 11:0 3OH | 0.867 | -0.149 | | -0.099 |
| *i*13:0 | -0.223 | 0.693 | | -0.574 |
| *a*13:0 | -0.288 | 0.727 | | -0.203 |
| 13:0 | -0.205 | 0.874 | | -0.365 |
| *i*12:0 3OH | -0.389 | -0.218 | | 0.195 |
| 12:0 3OH | 0.835 | 0.110 | | 0.323 |
| *i*14:0 | -0.174 | 0.757 | | -0.333 |
| 14:0 | 0.906 | -0.150 | | -0.106 |
| *i*15:1 G | 0.850 | -0.223 | | -0.128 |
| *a*15:1 A | 0.670 | 0.533 | | 0.216 |
| *i*15:0 | 0.810 | 0.344 | | 0.098 |
| *a*15:0 | -0.728 | -0.405 | | -0.045 |
| 15:1ω6*c* | 0.953 | -0.192 | | -0.188 |
| 15:1ω8*c* | 0.869 | -0.202 | | -0.235 |
| *i*16:1 G | 0.954 | -0.190 | | -0.177 |
| 16:0 | -0.855 | -0.059 | | 0.067 |
| *a*16:0 | 0.818 | 0.530 | | 0.100 |
| 16:1ω9*c* | 0.826 | 0.352 | | 0.006 |
| 16:1ω7*c*/16:1ω6*c* | -0.308 | 0.044 | | 0.767 |
| 16:1ω5*c* | 0.955 | -0.187 | | -0.178 |
| 16:0 | 0.960 | 0.092 | | 0.214 |
| 10*Me*16:0 | -0.813 | -0.447 | | -0.019 |
| *a*17:1 B/*i* I | 0.938 | 0.149 | | 0.258 |
| *a*17:1 A | 0.913 | 0.057 | | 0.019 |
| *i*17:0 | 0.955 | -0.188 | | -0.172 |
| *a*17:0 | 0.953 | -0.066 | | 0.111 |
| 17:1ω7*c* | 0.954 | -0.189 | | -0.174 |
| 17:1ω8*c* | 0.867 | 0.419 | | -0.034 |
| *cy*17:0 | 0.959 | -0.104 | | -0.128 |
| 17:0 | 0.871 | 0.467 | | 0.045 |
| 16:1 2OH | 0.608 | -0.250 | | -0.288 |
| 10*Me*17:0 | 0.966 | -0.081 | | 0.072 |
| *i*18:0 | 0.848 | -0.021 | | 0.200 |
| *a*18:0/18:2ω6,9*c* | 0.935 | 0.012 | | -0.039 |
| 18:1ω9*c* | 0.954 | -0.187 | | -0.309 |
| 18:1ω7*c*/18:1ω6*c* | 0.869 | -0.200 | | -0.230 |
| 18:1ω5*c* | -0.812 | -0.449 | | -0.011 |
| 18:0 | 0.922 | 0.171 | | 0.300 |
| 11*Me*18:1ω7*c* | 0.952 | -0.191 | | -0.185 |
| 10*Me*18:0, TBSA | -0.239 | 0.165 | | 0.713 |
| *i*19:1I | 0.953 | -0.067 | | 0.107 |
| 17:0 3OH | 0.931 | 0.154 | | 0.275 |
| *cy*19:0ω10*c*/19ω6 | -0.954 | -0.187 | | -0.171 |
| *cy*19:0ω8*c* | 0.951 | 0.099 | | 0.228 |
| 19:0 | 0.907 | 0.187 | | 0.329 |
| 18:0 2OH | 0.934 | 0.150 | | 0.268 |
| 20:4ω6,9,12,15*c* | 0.601 | -0.185 | | -0.298 |
| *i*20:0 | 0.118 | 0.547 | | 0.739 |
| 20:1ω9*c* | 0.650 | -0.089 | | -0.007 |
| 20:0 | -0.302 | -0.173 | | -0.332 |
